# Diabetes Medical Group Visits and Type 2 Diabetes Outcomes: Mediation Analysis of Diabetes Distress

**DOI:** 10.2196/57526

**Published:** 2025-02-06

**Authors:** Matthew Reichert, Barbara A De La Cruz, Paula Gardiner, Suzanne Mitchell

**Affiliations:** 1 Committee on Degrees in Social Studies, Harvard University, William James Hall, 3rd Floor, 33 Kirkland Street, Cambridge, MA, 02138, United States, 1 6319441975; 2 Department of Family Medicine and Community Health, University of Massachusetts Chan Medical School, Worcester, MA, United States

**Keywords:** diabetes, diabetic, diabetes mellitus, DM, type 1 diabetes, type 2 diabetes, diabetes mellitus type 2, diabetes outcomes, diabetes medical group visit, DMGVs, psychosocial functioning, psychosocial, glycemic control, glycemic, shared medical appointments, self-management, mediation analysis, social support, minority women, minority

## Abstract

**Background:**

Group-based diabetes care, both technology-enabled and in-person, can improve diabetes outcomes in low-income minority women, but the mechanism remains unclear.

**Objective:**

We tested whether diabetes group medical visits (GMVs) reduced hemoglobin A_1c_ (HbA_1c_) by mitigating diabetes distress (DD), an emotional response affecting nearly half of adults with type 2 diabetes in community settings.

**Methods:**

We conducted a mediation and moderation analysis of data from the Women in Control 2.0 comparative effectiveness study, which showed that both technology-enabled and in-person diabetes GMVs improve HbA_1c_. We tested whether DD mediated the relationship between diabetes GMV engagement and reductions in HbA_1c_. We also tested whether this relationship was moderated by depressive symptoms and social support. Participants were 309 low-income and minority women. Diabetes GMV engagement was measured using the Group Climate Questionnaire. The mediator, DD, was measured using the Diabetes Distress Screening Scale. The outcome was the 6-month change in HbA_1c_. Social support was measured using the Medical Outcomes Study Social Support Survey.

**Results:**

DD mediated the relationship between engagement and 6-month HbA_1c_. Specifically, group engagement affected HbA_1c_ by reducing distress associated with the regimen of diabetes self-management (*P*=.04), and possibly the emotional burden of diabetes (*P*=.09). The relationship between engagement and 6-month HbA_1c_ was moderated by depressive symptoms (*P*=.02), and possibly social support (*P*=.08).

**Conclusions:**

Engagement in diabetes GMVs improved HbA_1c_ because it helped reduce diabetes-related distress, especially related to the regimen of diabetes management and possibly related to its emotional burden, and especially for women without depressive symptoms and possibly for women who lacked social support.

## Introduction

Over 37 million people in the United States live with type 2 diabetes mellitus (T2DM), accounting for 7.8 million hospitalizations and over US $327 billion in health care costs annually, with persistent disparities in diabetes outcomes among low-income and minority adults being attributable to underlying health inequities [1-7]. Unmet social needs, such as housing, job, and food insecurity and structural barriers to health care, among them inadequate access, affordability, and quality make it difficult for underserved communities to access the medical care and support needed to effectively manage diabetes, increasing the burden of living with chronic disease for this segment of the population [8].

The overwhelming stress of diabetes self-management can produce an emotional response characterized as diabetes distress (DD). A distinct psychological consequence of living with T2DM, DD is more common than comorbid depression and anxiety, with prevalence estimates ranging from 36% to 45% [9-11]. It has been linked to poor glycemic control, self-management, and self-efficacy among adult patients [12-15]. DD is a treatable barrier to effective diabetes self-management that is gaining increasing attention in primary and specialty care. A 2017 position paper from the American Diabetes Association recommended routine screening and integration of psychosocial care, considering emotional status and presence of a social support network, to improve the treatment course of those living with T2DM [9,16].

Identifying scalable approaches that address both the physical and mental health needs of those living with diabetes is a high priority. Emerging research has shown that group-based diabetes care can lead to positive health outcomes. Group-based education is often promoted as an effective approach to managing type 2 diabetes, with the potential to enhance self-management skills and improve health outcomes [17]. An alternative to individual clinical encounters, diabetes group medical visits (GMVs) convene groups of patients to receive peer support, diabetes self-management education, and a clinical consult within the context of a 2-hour shared appointment [18,19]. There is substantial published evidence demonstrating the clinical effectiveness of standard, in-person diabetes GMVs (or shared medical appointments) compared to usual care for adults living with diabetes. Four systematic reviews conclude that diabetes GMVs are clinically supported for improving glycemic control [18-21]. This GMV model of care has been associated with improved self-management mastery, quality of life, and mental health [18,19]. It can also reduce health disparities by fostering more equitable patient-provider relationships, creating relationships of care between patients, and improving health literacy [22]. However, implementing group-based care is not without challenges given heterogeneity of implementation across busy clinical practices, particularly those serving low-income and diverse communities and limited reporting [17,21,23].

Health technologies may bridge gaps in access to effective models of diabetes care, such as diabetes GMVs, but research on the effectiveness and scalability of existing applications is limited. In the Women in Control 2.0 (WIC2) study, our team tested the effectiveness of virtual, technology-enabled diabetes GMVs versus in-person GMVs for low-income, English- and Spanish-speaking minority women with uncontrolled diabetes [24]. Our findings showed that GMVs, whether in-person or technology-enabled, improved not only 6-month hemoglobin A_1c_ (HbA_1c_), but also 6-month DD. For this reason, we hypothesized that DD may mediate the effect of GMVs on glucose control. We further hypothesized that group-based care reduced DD by cultivating a sense of belonging, an opportunity to feel connected, heard, and understood by other participants with lived experience managing diabetes. The intervention, methods, and main results from the WIC2 study are reported elsewhere [24-26].

**Figure 1.**
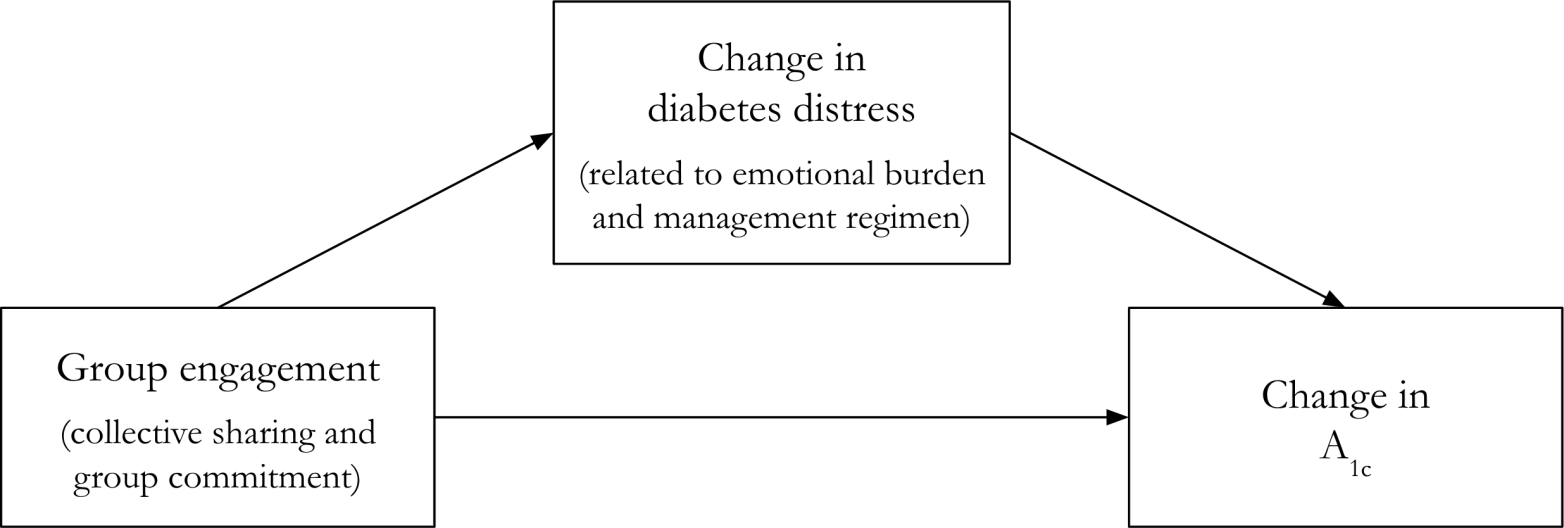
Conceptual model. A_1c_: hemoglobin A_1c_.

To test this conceptual model, we conducted a mediation analysis substudy of clinical trial data from the WIC2 study to determine whether participants’ self-reported engagement with other group members affected glucose control by reducing DD or its subcomponents (Figure 1). We also aimed to test whether baseline characteristics moderated the relationship between engagement and HbA_1c_.

## Methods

### Study Design

The WIC2 noninferiority, randomized controlled trial compared over-time changes in HbA_1c_ among 309 women randomly assigned to attend either in-person or technology-enabled GMVs, both led by a prescribing clinician and a trained facilitator for 8 weeks and delivered in English or Spanish, depending on participants’ language preferences at baseline. All participants then entered a 16-week maintenance period during which no GMVs took place, but participants were instructed to self-monitor nutrition and physical activity. Of 309 randomized participants, 207 (67%) met per-protocol criteria by attending 6 of 8 sessions. Noninferior improvements were detected in mean HbA_1c_ from baseline to 6 months in both groups: HbA_1c_ declined by −0.7% (SD 1.8%) among participants attending in-person GMV and by −0.5% (SD 1.6%) among participants attending virtual world GMV (*P*<.001) [23,24].

This WIC2 secondary analysis tested whether the improvements in HbA_1c_ observed in the WIC2 study were associated with group engagement, whether this occurred through lowering DD, and whether that relationship was conditional on the following moderators measured at baseline: language, health literacy, depressive symptoms, anxiety, patient activation, HbA_1c_, and social support. These analyses included all participants, irrespective of meeting per-protocol criteria by attending at least 6 sessions.

### Mediation

The explanatory variable, group engagement, was measured using the group engagement subscale of the Group Climate Questionnaire (GCQ-S)—a validated survey completed at baseline, 9 weeks, and 6 months assessing group cohesion [27]. Group cohesion has been conceptualized as 2 domains: affective, which is associated with the individual’s attraction to the group or its members and ability to collectively share positive, as well as negative, emotional experiences; and behavioral, a domain associated with the individual’s sense of commitment to the group [28,29]. The engagement subscale of group cohesion captures both these collective sharing and group commitment domains.

Each question from the GCQ-S was scored from 0 (“not at all”) to 6 (“extremely”). A total score was determined by calculating the mean response to questions from the 5 items of the group engagement subscale, shown in Table S1 in Multimedia Appendix 1.

The potential mediators, self-reported DD and its subcomponents, were collected using the Diabetes Distress Screening Scale (DDS-17) at baseline, 9 weeks, and 6 months [10,30]. The subscales for the DDS-17 assess the emotional burden of diabetes, regimen of diabetes management, perceived quality of diabetes care from a physician, and interpersonal support from family and friends. We hypothesized that group engagement influenced HbA_1c_ primarily by reducing distress associated with the emotional burden and regimen of diabetes management, because these were most directly targeted by the peer support and self-management components of the WIC2 curriculum in GMVs. We did not expect that GMVs would directly impact DD related to care from a physician and interpersonal support from family and friends.

Each question on the DDS-17 was scored from 1 (“not a problem”) to 6 (“a very serious problem”) and is listed in Table S1 in Multimedia Appendix 1. The total DD and subscale scores were calculated by taking the mean of all scale and subscale scores.

### Moderation

We also tested whether baseline social support, Spanish as a primary language, health literacy, depressive symptoms, anxiety, patient activation, or HbA_1c_ moderated the relationship between group engagement and the 6-month change in HbA_1c_.

Because the GMVs were group-based, we expected that they would be particularly helpful for participants who did not already enjoy supportive social networks. To measure social support, we used the Medical Outcomes Study Social Support Survey, a 19-item instrument developed for a 2-year study of patients with chronic conditions. The instrument has 4 subscales capturing emotional or informational, tangible, affectionate, and positive social interaction-related social support [31] (see Table S1 in Multimedia Appendix 1).

We also expected health literacy and patient activation to magnify the effect of group engagement by helping participants take fuller advantage of the WIC2 curriculum. High baseline anxiety or depressive symptoms may dampen the effect of group engagement by compounding the emotional or regimen-related burden of DD. Low baseline HbA_1c_ may produce ceiling effects. Finally, we checked for differences across the culturally equivalent Spanish- and English-language WIC2 curricula.

### Statistical Analyses

To identify potential confounders, participants with low group engagement (≤median score) versus high engagement (>median score) were compared on baseline characteristics of the sample with means and SDs or percentages.

To summarize the main outcome variables and potential mediators, we took baseline and 6-month means and SDs as well as mean changes over time with SDs. We performed paired *t* tests on baseline versus 6-month values.

We tested whether the relationship between group engagement and HbA_1c_ was mediated by DD or its subscores in two ways. First, we performed a series of ordinary least squares (OLS) regressions. We regressed the primary outcome (6 mo change in HbA_1c_) on the explanatory variable (group engagement), the outcome (6 mo change in HbA_1c_) on the potential mediators (DD and each of its subscales), and the potential mediators (DD and each of its subscales) on the explanatory variable (group engagement). For each, we ran both a bivariate regression and a multivariate regression that included cohort fixed effects and controlled for study arm.

Second, we performed mediation by simulation, using the *mediation* package for R (R Foundation) [32,33]. Using this method, we estimated the average causal mediation effect. As this is a secondary analysis that was not originally powered with causal mediation in mind, we expect this method to underestimate any true mediated effect.

Finally, we used OLS regression to determine whether Spanish as a primary language, health literacy, depressive symptoms, anxiety, patient activation, baseline HbA_1c_, or social support and its subscores moderated the relationship between group engagement and 6-month change in HbA_1c_. We regressed the 6-month change in HbA_1c_ on group engagement interacted with each potential moderator. As with mediation by simulation, due to sample size, we expect this to be a conservative estimate of moderated effects.

### Ethical Considerations

Informed consent and approval by the Boston University or Boston Medical Center Institutional Review Board (H-34220) are documented in the WIC2 study [24]. All eligible and interested participants were consented and enrolled abiding by the principles of the Belmont Report and the Declaration of Helsinki. The informed consent process included a teach-back approach by which participants’ understanding of this study’s procedures, risk or benefits, and voluntary nature was confirmed. Enrolled participants self-reported their answers to research surveys about their health and lived experience with diabetes. All research data were stored in password-protected, HIPAA (Health Insurance Portability and Accountability Act)-compliant systems and linked with a study-generated identifier to protect confidentiality.

## Results

### Description of the Sample

A full description of the WIC2 study population was previously published [24]. In brief, participants’ mean age was 56 (SD 10.4) years and mean HbA_1c_ was 9.93% (SD 1.74%). All participants were female (n=309), 63.1% (195/309) self-identified as Black or African American, while 23.6% (73/309) were Spanish-speaking. A majority of participants (70.9%, 219/309) reported Medicaid, Medicare, or both as their insurance provider. Fifteen percent (47/309) of participants reported an anxiety disorder, and 25.2% (78/309) of participants reported a depressive disorder, including depression, major depression, dysthymia, or minor depression. Mean total DD was 2.27 (maximum score of 6; SD 1.04). See Table 1 for the mean DD subscales. No apparent differences were detected between low-engagement and high-engagement participants on observed characteristics. Remaining characteristics are summarized in Table 1.

**Table 1.** Baseline sample characteristics for all participants and participants with above versus below median group engagement.

Characteristics		Total (N=309)	Engage^a^ ≤ median (3.8; n=123)	Engage >median (3.8; n=114)
Spanish-speaking, n (%)		73 (24)	30 (24)	29 (25)
Low health literacy, n (%)		87 (28)	36 (29)	33 (29)
Anxiety disorder, n (%)		47 (15)	16 (13)	19 (17)
Depressive disorder^b^, n (%)		78 (25)	29 (24)	32 (28)
PAM-13^c^, mean (SD)		66.12 (20.56)	66.1 (19.47)	69.31 (19.05)
**Social support^d^** **, mean (SD)**				
	Overall	3.78 (1.06)	3.68 (1.09)	3.9 (1.02)
	Affectionate	4.05 (1.11)	3.93 (1.16)	4.17 (1.06)
	Emotional or informational	3.82 (1.11)	3.71 (1.16)	3.96 (1.06)
	Positive social interaction	3.80 (1.2)	3.75 (1.2)	3.91 (1.18)
	Tangible	3.51 (1.26)	3.43 (1.23)	3.58 (1.3)
**Diabetes distress^e^** **, mean (SD)**				
	Total DD^f^	2.27 (1.04)	2.22 (1.08)	2.36 (1.03)
	Regimen DD	2.64 (1.33)	2.56 (1.36)	2.82 (1.34)
	Emotional burden DD	2.69 (1.44)	2.61 (1.47)	2.81 (1.5)
	Physician DD	1.53 (0.99)	1.45 (0.94)	1.56 (1.02)
	Interpersonal DD	1.97 (1.28)	2.05 (1.45)	1.89 (1.12)
HbA_1c_^g^, mean (SD)		9.93 (1.74)	9.74 (1.65)	10.05 (1.86)
Age, mean (SD)		55.62 (10.4)	56.17 (10.1)	53.94 (10.55)
**Race, n (%)**				
	Black or African American	195 (63)	81 (66)	76 (67)
	White	26 (8)	12 (10)	11 (10)
	Other race	78 (25)	30 (24)	27 (24)
**Hispanic, n (%)**				
	Yes	105 (35)	41 (33)	40 (35)
	No	195 (65)	82 (66)	74 (65)
**Insurance, n (%)**				
	Commercial	69 (22)	28 (23)	29 (25)
	Medicare or Medicaid	219 (71)	88 (72)	82 (72)
**Education, n (%)**				
	High school graduate or less	152 (49)	63 (51)	54 (47)
	Any college, vocational, or trade school	132 (43)	53 (43)	53 (46)
	Any postgraduate	14 (5)	6 (5)	7 (6)
**Employment status, n (%)**				
	Full-time	75 (24)	28 (23)	35 (31)
	Part-time	44 (14)	19 (15)	16 (14)
	Not employed	156 (50)	68 (55)	51 (45)
**Household income, n (%)**				
	≤US $29,999	140 (45)	51 (41)	56 (49)
	≥US $30,000	59 (19)	25 (20)	23 (21)
	Refused, do not know, or none	101 (33)	47 (38)	35 (31)

a Assessed using the engagement subscale of the Group Climate Questionnaire (GCQ-S).

b Including depression, major depression, dysthymia, or minor depression.

c PAM-13: Patient Activation Measure.

d Assessed using the Medical Outcomes Study Social Support Survey.

e Assessed using the Diabetes Distress Screening Scale (DDS-17).

f DD: diabetes distress.

g HbA_1c_: hemoglobin A_1c_.

### Results of Main Relationships

The outcome, HbA_1c_, decreased from 9.9% (SD 1.7) at baseline to 9.3% at 6 months (SD 2) on average (*P*<.001, via paired 2-tailed *t* test). The potential mediators—total DD score and each DD subscore—also decreased from baseline to 6 months (*P*<.001 for all DD scores except the physician subscore [*P*=.095, via paired *t* test]). The magnitude of this decrease was greatest for the regimen (−0.6, SD 1.2) and emotional burden subscores (−0.6, SD 1.2; Table 2).

**Table 2.** Summary of main outcome variables and potential mediators (all participants).

	Baseline, mean (SD)	6 Months, mean (SD)	Change, mean (SD)	*P* value^a^
Group engagement^b^	N/A^c^	3.6 (1.3)	N/A	N/A
Diabetes distress^d^	2.3 (1)	1.9 (1)	−0.4 (0.9)	<.001
DD^e^ regimen	2.6 (1.3)	2.1 (1.2)	−0.6 (1.2)	<.001
DD emotional burden	2.7 (1.4)	2.2 (1.3)	−0.6 (1.2)	<.001
DD physician	1.5 (1)	1.4 (0.9)	−0.1 (1)	.095
DD interpersonal	2 (1.3)	1.7 (1.2)	−0.3 (1.2)	<.001
Hemoglobin A_1c_	9.9 (1.7)	9.3 (2)	−0.6 (1.7)	<.001

a *P* value from a paired 2-tailed *t* test.

b Assessed using the engagement subscale of the Group Climate Questionnaire (GCQ-S).

c N/A: not applicable.

d Assessed using the Diabetes Distress Screening Scale (DDS-17).

e DD: diabetes distress.

**Figure 2.**
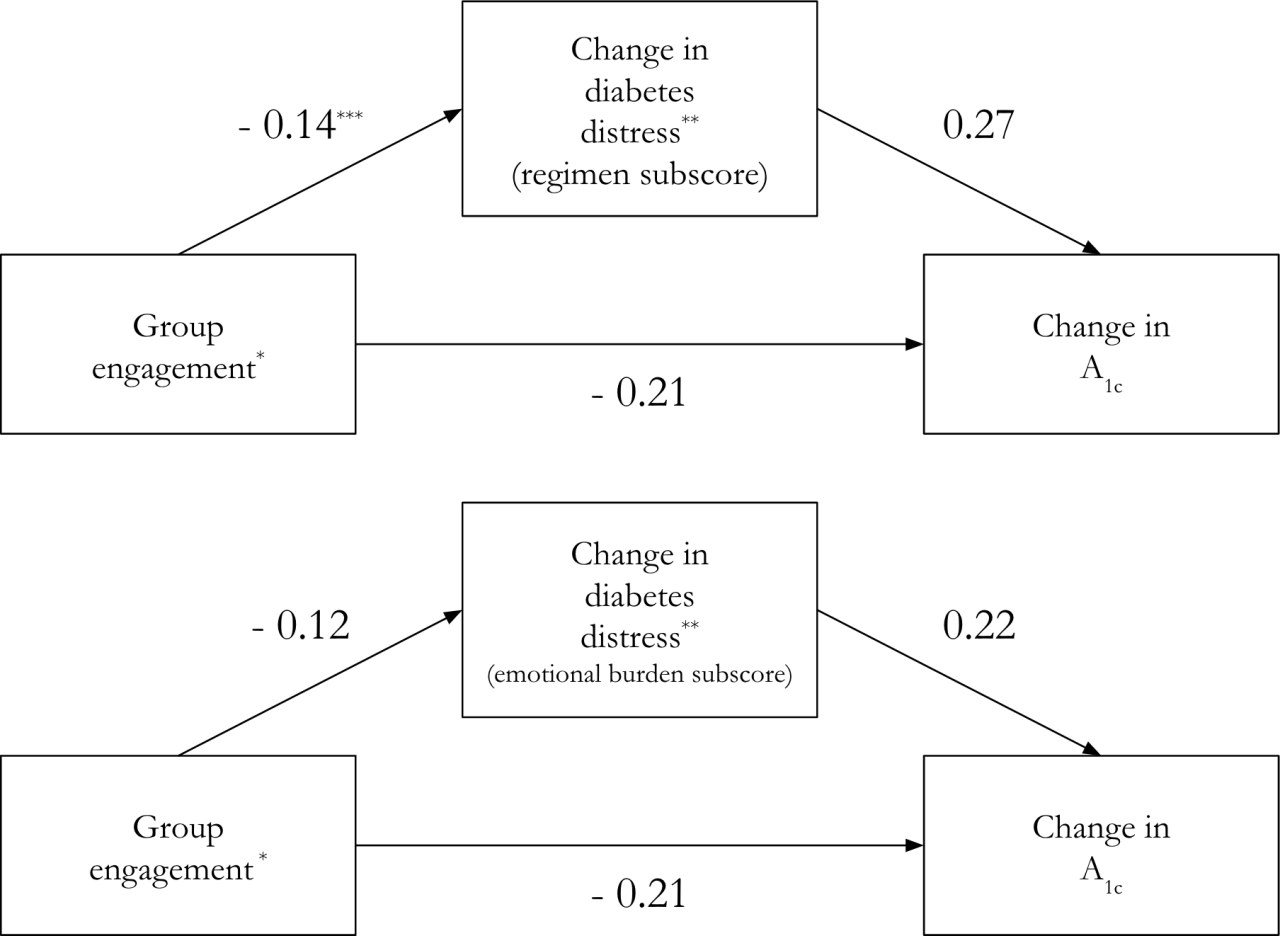
Coefficients on mediator relationships of interest from OLS regressions ^*^ Assessed using the engagement subscale of the Group Climate Questionnaire (GCQ-S). ** Assessed using regimen and emotional burden subscales of the Diabetes Distress Screening Scale (DDS-17). *** Coefficients and *P* value thresholds derived from Table 3 OLS regressions. DDS-17: Diabetes Distress Screening Scale; GCQ-S: Group Climate Questionnaire; OLS: ordinary least square.

Table 3 summarizes the individual associations between the outcome, mediators, and independent variable, and Figure 2 maps those associations to our conceptual model.

**Table 3.** Main relationships between outcome, explanatory variables, and mediators.

	Bivariate^a^		Fixed effects^b^	
	Coefficient (SE)	*P* value	Coefficient (SE)	*P* value
HbA_1c_^c^ on engagement^d^	−0.21 (0.08)	.01^d^	−0.25 (0.08)	.004^d^
Distress (total)^e^ on engagement	−0.1 (0.04)	.03^d^	−0.1 (0.05)	.03^d^
Distress (regimen) on engagement	−0.14 (0.06)	.02^d^	−0.16 (0.06)	.01^d^
Distress (emotional burden) on engagement	−0.12 (0.06)	.04^d^	−0.12 (0.06)	.04^d^
Distress (physician) on engagement	−0.1 (0.05)	.04^d^	−0.08 (0.05)	.011^d^
Distress (interpersonal) on engagement	0 (0.06)	.94	0.01 (0.06)	.90
HbA_1c_ on distress (total)	0.24 (0.12)	.048^d^	0.24 (0.12)	.04
HbA_1c_ on distress (regimen)	0.27 (0.09)	.002	0.26 (0.09)	.004
HbA_1c_ on distress (emotional burden)	0.22 (0.09)	.02^d^	0.2 (0.09)	.03^d^
HbA_1c_ on distress (physician)	0 (0.11)	.996	0.04 (0.11)	.74
HbA_1c_ on distress (interpersonal)	−0.02 (0.09)	.84	0 (0.09)	.98

a Ordinary least square regression, described in left-hand column.

b Ordinary least square regression, controlling for study arm and with cohort fixed effects, described in left-hand column.

c HbA_1c_: hemoglobin A_1c_.

d Assessed using the engagement subscale of the Group Climate Questionnaire (GCQ-S).

e Assessed using the Diabetes Distress Screening Scale (DDS-17).

We detected a negative relationship between group engagement score and 6-month change in HbA_1c_. A one-point increase in group engagement score was associated with, on average, a 0.21 greater decrease in HbA_1c_ from baseline to 6 months. This was true both without (*P*=.01) and with (*P*=.004) cohort fixed effects and controlling for study arm.

In Table 3, we also detected a negative relationship between group engagement and all DD mediators, except for the interpersonal subscore. A one-point increase in group engagement score was associated with, on average, a 0.1 greater decrease in total DD score from baseline to 6 months (*P*=.03), a 0.14 greater decrease in regimen subscore (*P*=.02), a 0.12 greater decrease in emotional burden subscore (*P*=.04), and a 0.1 greater decrease in physician subscore (*P*=.04). The results were similar with and without cohort fixed effects and controlling for study arm.

Finally, we detected a positive relationship between 3 mediators and 6-month change in HbA_1c_: total DD, and the regimen and emotional burden subscores. A one-point decrease in the regimen subscore was associated with, on average, a 0.27% greater decrease in HbA_1c_ from baseline to 6 months, again both without (*P*=.002) and with (*P*=.004) cohort fixed effects and controlling for study arm. A one-point decrease in the emotional burden subscore was associated with, on average, a 0.22% greater decrease in the change in HbA_1c_ from baseline to 6 months, both without (*P*= .02) and with (*P*=.03) cohort fixed effects and controlling for study arm.

### Results of Mediator Analysis

Table 4 lists the total effect of engagement on the 6-month change in HbA_1c_, the average causal mediation effect (the proportion of the total effect that runs through the mediator), and the average direct effect (the remaining proportion of the total effect that does not run through the mediator), calculated by simulation, for each of five possible mediators: DD and each of its 4 subscores.

**Table 4.** Mediator analysis^a^.

Mediator	Total effect	*P* value	ADE^b^	*P* value	ACME^c^	*P* value
Diabetes distress (total)^d^	−0.2	.02^a^	−0.18	.026^a^	−0.02	.20
Distress (regimen)	−0.2	.02^a^	−0.16	.048^a^	−0.04	.04^a^
Distress (emotional burden)	−0.2	.02^a^	−0.18	.042^a^	−0.02	.09
Distress (physician)	−0.2	.01^a^	−0.2	.014^a^	0	.798
Distress (interpersonal)	−0.2	.02^a^	−0.2	.02^a^	0	.92

a Mediation by simulation performed using *mediate* package in R.

b ADE: average direct effect.

c ACME: average causally mediated effect.

d Assessed using the Diabetes Distress Screening Scale (DDS-17).

An average causally mediated effect of group engagement on 6-month change in HbA_1c_ was detected that runs through the regimen (*P*=.04) of DD. An average causally mediated effect of group engagement on 6-month change in HbA_1c_ may also run through the emotional burden of DD (*P*=.094).

There was no evidence that total DD mediated the relationship between group engagement and 6-month change in HbA_1c_ (*P*=.20). There was also no evidence that the physician (*P*=.798) or interpersonal (*P*=.92) DD subscores mediated this relationship.

### Results of Moderator Analyses

**Figure 3.**
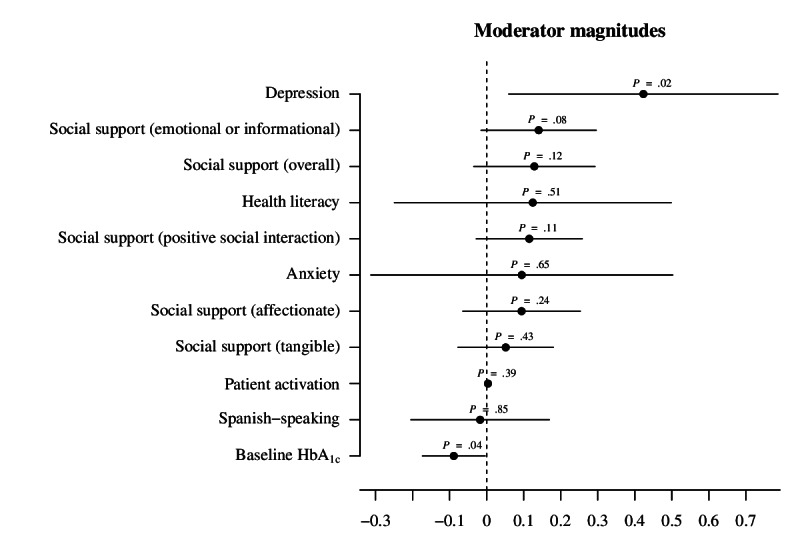
Moderator effects are plotted as coefficients on OLS model interaction terms with 95% CIs. *P* values are for each OLS model interaction term. Social support and subscores were assessed using the Medical Outcomes Study Social Support Survey. Health literacy was assessed with the yes or no question “Do you usually ask someone to help you read materials you receive from the hospital?” Patient activation was assessed using PAM-13. Depression includes depression, major depression, dysthymia, or minor depression. OLS: ordinary least square; PAM-13: Patient Activation Measure.

Figure 3 plots coefficients with 95% CIs from the interaction terms of each OLS model regressing 6-month change in HbA_1c_ on engagement interacted with the potential moderators. Baseline depressive symptoms, emotional or informationally based social support, and baseline HbA_1c_ were found to moderate the relationship between group engagement and 6-month change in HbA_1c_. Participants that did not report depression, major depression, dysthymia, or minor depressive symptoms at baseline saw their HbA_1c_ decline by an additional 0.42% for each one-point increase in group engagement score (*P*=.02). For each lower point of self-reported emotional or informationally based social support, participants saw their HbA_1c_ decline by an additional 0.14% for each one-point increase in group engagement score (*P*=.08), though a larger sample size is needed to confirm this result. For each additional percentage point of baseline HbA_1c_, participants saw their 6-month HbA_1c_ decline by an additional 0.09% with each one-point increase in group engagement score (*P*=.04).

## Discussion

### Summary of Findings

While GMVs are associated with improved glucose control, the underlying mechanism of how group-based care is linked to improved outcomes has been unclear. This analysis of mediators provides evidence that engaging in GMVs (either in-person or technology-enabled) works to lower HbA_1c_, in part, by reducing the components of DD associated with the management regimen of diabetes, and possibly also the emotional burden of diabetes management.

Specifically, we found that while the regimen and possibly the emotional burden components of DD mediated the effect of GMVs, the physician or interpersonal (with family or friends) components of DD did not. The mediated effect for total DD, measured as a summary score from the DDS-17, was not significant (*P*=.20), and was likely diluted by the components of total DD making up the physician and interpersonal subscores.

These findings are consistent with our hypothesis that GMVs target a participant’s ability to self-manage diabetes and, possibly, cultivate a sense of belonging and shared understanding by relating to others within the group. In particular, GMVs may improve regimen-related DD by alleviating the stigma of failing in self-management behaviors, fostering peer-supported adherence to treatment, and improving health literacy. GMVs likely target emotional burden-related DD by building psychological safety, providing social acceptance, and mitigating feelings of powerlessness. This is also consistent with findings from the DDS-17 developers that the regimen and emotional burden distress subscales contribute most significantly to the total DD [34].

These findings also suggest that GMVs may be less relevant for how participants relate to their broader social networks outside the group, such as friends, family, and physicians. Support from peers specifically within the GMVs may be key to the relationship between GMV engagement, improved DD, and improved glycemic control, as previous studies have also found that peer-to-peer social, emotional and informational support, both with and without technology supplement, can improve glycemic control and reduce DD among minority groups [35-38].

Our moderation analysis showed that engagement in group visits was most strongly associated with decline in HbA_1c_ for participants with higher baseline HbA_1c_, without depressive symptoms at baseline, and, possibly, who reported little emotional or informationally based social support.

Participants that reported low emotional and informational social support may have especially benefited from GMVs that offered an empathetic social setting that they may have otherwise lacked, though a larger sample size is required to confirm this result.

In contrast, participants with comorbid depressive symptoms may have struggled with practicing the self-management behaviors prescribed in the GMVs. Existing research has also found that depressive symptoms can inhibit self-management mastery and undermine treatment focused on diabetes empowerment [39,40]. Individuals who feel they have little control over their T2DM and are unable to reach treatment goals report less motivation to manage their condition [41]. In light of studies showing that DD, but not depressive symptoms by themselves, have a concurrent and longitudinal association with HbA_1c_ levels, these findings suggest that comorbid depressive symptoms may negatively influence HbA_1c_ primarily by rendering diabetes self-management education and support less effective [12].

### Limitations

First, these analyses tested mediators of group engagement, rather than a direct measure of the intervention. Testing for a mediator of the study arm was not possible because these data were generated by a noninferiority trial that, by design, randomized participants to 2 interventions that both improved HbA_1c_. As technology-enabled GMVs were noninferior to their in-person counterparts, the study arm by itself does not generate meaningful variation on the explanatory variable. Furthermore, testing for an effect of intervention adherence sacrifices sample size, as few participants had substantially low attendance. Engagement offered the variation on the explanatory variable while still representing a meaningful measure of participation in GMVs. In the absence of validated standalone measures of engagement for group interventions, we used the engagement subcomponent of the GCQ-S. Nevertheless, we did replicate our mediation analysis using the study arm, and these results are summarized in Table S2 in Multimedia Appendix 1.

Second, this was a secondary analysis of data from the existing, published WIC2 study, which was not originally powered to detect mediation or moderation. This biases us toward type II error (false negatives), or against detecting a mediated or moderated effect even where one may exist. In practice, our sample size can support the simple OLS regressions we use in our first mediation analysis (Table 3 and Figure 2), but may be too small for more complex analysis such as mediation by simulation (Table 4) and interaction effects (Figure 3). For this reason, in addition to reporting findings where *P*<.05, we also report findings for *P* values lower than 0.1 and interpret them as suggestive of relationships that we might detect given a larger sample. In particular, our analyses may underestimate the role of the emotional burden of DD as a mediator; while our mediation analysis using regression did detect a mediation effect for the emotional burden of DD in models both with and without controls and cohort fixed effects, our mediation analysis using simulation can only suggest this at *P*=.09.

Third, while this study detected an average causally mediated effect of regimen-related and emotional burden-related DD, it also estimated an average direct effect that runs through other mediators. Specifically, regimen-related and emotional burden-related DD were found to mediate 30% of the total effect of engagement on HbA_1c_, leaving 70% of the effect, which runs through other mediators, to be explained in further research.

Finally, because group engagement was not randomly assigned, though no observed confounding was detected, this study cannot rule out unobserved confounding on the relationship between engagement and DD or on the relationship between DD and HbA_1c_.

### Conclusions

Our findings showed that engagement in group-based diabetes care improved HbA_1c_ by way of reducing diabetes-related distress, especially the components related to the regimen and possibly the emotional burden of living with T2DM. Strategies that encourage collective sharing and group commitment should be actively integrated in GMVs to positively influence diabetes outcomes such as DD and glucose control. Additionally, it is important to identify patients with comorbid depressive symptoms and, possibly, those lacking social support separate from the GMVs, as our findings confirmed previous research suggesting that untreated depressive symptoms may interfere with the positive effects of medical group-based care [39,40]. Future research should explore how care models can be more effective in specifically treating patients with depressive symptoms and other comorbid conditions.

## Supplementary material

10.2196/57526 Multimedia Appendix 1 Group cohesion, diabetes distress, and social support instruments; relationships with this study’s treatment; full group cohesion measure; and moderator predicted values.

## Abbreviations

DD: diabetes distress
DDS-17: Diabetes Distress Screening Scale
GCQ-S: Group Climate Questionnaire
GMV: group medical visit
HbA_1c_: hemoglobin A_1c_
HIPAA: Health Insurance Portability and Accountability Act
OLS: ordinary least square
T2DM: type 2 diabetes mellitus
WIC2: Women in Control 2.0
