# Supplementary material for: Diabetes Medical Group Visits and Type 2 Diabetes Outcomes: Mediation Analysis of Diabetes Distress
Source: JMIR Diabetes. 2025 Feb 6;10:e57526. doi: 10.2196/57526 (PMC11825897; doi:10.2196/57526)
Supplement: Multimedia Appendix 1 [file diabetes-v10-e57526-s001.docx]

**Table S1.** Group cohesion, diabetes distress, and social support instruments.

| **Group Climate Questionnaire (GCQ-S), Engagement Subcomponent and Questions** | |
| --- | --- |
|  |  |
| Engagement | The members liked and cared about each other |
|  | The members tried to understand why they do the things they do, tried to reason it out |
|  | The members felt what was happening was important and there was a senses of participation |
|  | The members challenged and confronted each other in their efforts to sort things out |
|  | The members revealed sensitive personal information or feelings |

| **Diabetes Distress Screening Scale (DDS-17),** **Subcomponents and Questions** | |
| --- | --- |
|  |  |
| Regimen | Feeling that I am not testing my blood sugars frequently enough |
|  | Feeling that I am often failing with my diabetes regimen. |
|  | Not feeling confident in my day-to-day ability to manage diabetes. |
|  | Feeling that I am not sticking closely enough to a good meal plan. |
|  | Not feeling motivated to keep up my diabetes self-management. |
|  |  |
| Emotional Burden | Feeling that diabetes is taking up too much of my mental and physical energy every day. |
|  | Feeling angry, scared, and/or depressed when I think about living with diabetes |
|  | Feeling that my diabetes controls my life |
|  | Feeling that I will end up with serious long-term complications, no matter what I do. |
|  | Feeling overwhelmed by the demands of living with diabetes. |
|  |  |
| Physician | Feeling that my doctor doesn't know enough about diabetes and diabetes care |
|  | Feeling that my doctor doesn't give me clear enough directions on how to manage my diabetes |
|  | Feeling that my doctor doesn't take my concerns seriously enough. |
|  | Feeling that I don't have a doctor who I can see regularly about my diabetes. |
|  |  |
| Interpersonal | Feeling that friends or family are not supportive enough of my self-care efforts |
|  | Feeling that friends or family don't appreciate how difficult living with diabetes can be. |
|  | Feeling that friends or family don't give me the emotional support that I would like. |

| **Medical Outcomes Study Social Support Survey, Subcomponents and Questions**    People sometimes look to others for companionship, assistance, or other types of support. How often is each of the following kinds of support available to you if you need it? | |
| --- | --- |
|  |  |
| Emotional/  Informational  Support | Someone you can count on to listen to you when you need to talk |
|  | Someone to give you information to help you understand a situation |
|  | Someone to give you good advice about a crisis |
|  | Someone to confide in or talk to about yourself or your problems |
|  | Someone whose advice you really want |
|  | Someone to share your most private worries and fears with |
|  | Someone to turn to for suggestions about how to deal with a personal problem |
|  | Someone who understands your problems |
|  |  |
| Tangible Support | Someone to help you if you were confined to bed |
|  | Someone to take you to the doctor if you needed it |
|  | Someone to prepare your meals if you were unable to do it yourself |
|  | Someone to help with daily chores if you were sick |
|  |  |
| Affectionate Support | Someone who shows you love and affection |
|  | Someone to love and make you feel wanted |
|  | Someone who hugs you |
|  |  |
|  |  |
| Positive Social Interaction | Someone to have a good time with |
|  | Someone to get together with for relaxation |
|  | Someone to do something enjoyable with |
| Additional Item | Someone to do things with to help you get your mind off things |

**Table S2.** Relationships with the study treatment.

| **Conceptual Model** | **ACME ^*^** | **P Value** |
| --- | --- | --- |
|  |  |  |
| Attendance → DD Regimen → A1c | -0.02 | 0.340 |
| Attendance → DD Emotional Burden → A1c | 0.00 | 0.800 |
| Attendance → Engagement → DD Regimen | -0.01 | 0.380 |
| Attendance → Engagement → DD Emotional Burden | -0.01 | 0.440 |
| Attendance → Engagement → A1c | -0.02 | 0.360 |
|  |  |  |
|  |  |  |
| Study Arm → DD Regimen → A1c | 0.00 | 1.000 |
| Study Arm → DD Emotional Burden → A1c | 0.04 | 0.260 |
| Study Arm → Engagement → DD Regimen | 0.04 | 0.094 |
| Study Arm → Engagement → DD Emotional Burden | 0.03 | 0.120 |
| Study Arm → Engagement → A1c | 0.05 | 0.160 |
|  |  |  |

^*^ Average Causal Mediated Effect

To test whether engagement or diabetes distress mediates the WIC2 intervention itself, using mediation by simulation we estimated the average causal mediation effect (ACME) for the following alternative conceptual models:

- DD regimen or emotional burden subscore mediates the relationship between attendance and A1C
- Engagement mediates the relationship between attendance and DD regimen or emotional burden subscore
- Engagement mediates the relationship between attendance and A1C
- DD regimen or emotional burden subscore mediates the relationship between study arm and A1C
- Engagement mediates the relationship between study arm and DD regimen or emotional burden subscore
- Engagement mediates the relationship between study arm and A1C

**Table S3.** Full group cohesion measure.

| **Mediator** | **Total Effect** | **P value** | **ADE ^*^** | **P value** | **ACME** ^†^ | **P value** |
| --- | --- | --- | --- | --- | --- | --- |
| Diabetes Distress (Total) | - 0.08 | 0.216 | - 0.07 | 0.230 | 0.00 | 0.598 |
| DD Regimen | - 0.08 | 0.204 | - 0.07 | 0.260 | - 0.01 | 0.412 |
| DD Emotional Burden | - 0.08 | 0.244 | - 0.07 | 0.300 | - 0.01 | 0.362 |
| DD Physician | - 0.08 | 0.214 | - 0.08 | 0.210 | 0.00 | 0.994 |
| DD Interpersonal | - 0.08 | 0.182 | - 0.08 | 0.192 | 0.00 | 0.904 |

^*^ Average Direct Effect

^†^ Average Causal Mediated Effect

To test whether our findings are sensitive to , using mediation by simulation we estimated the average causal mediation effect (ACME) for the effect on A1c, mediated by diabetes distress and its subscores, of the full GCQ-S group cohesion score, including not only engagement but also its conflict and avoidance subcomponents.
**Figure S1.** Moderator predicted values.


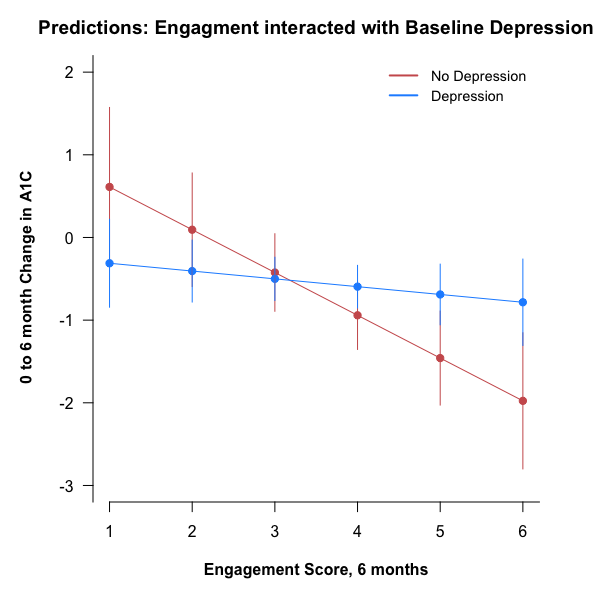

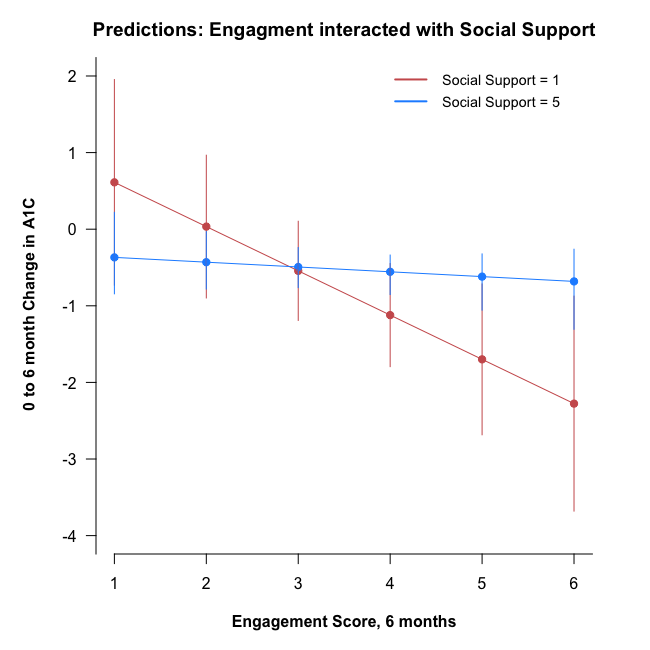


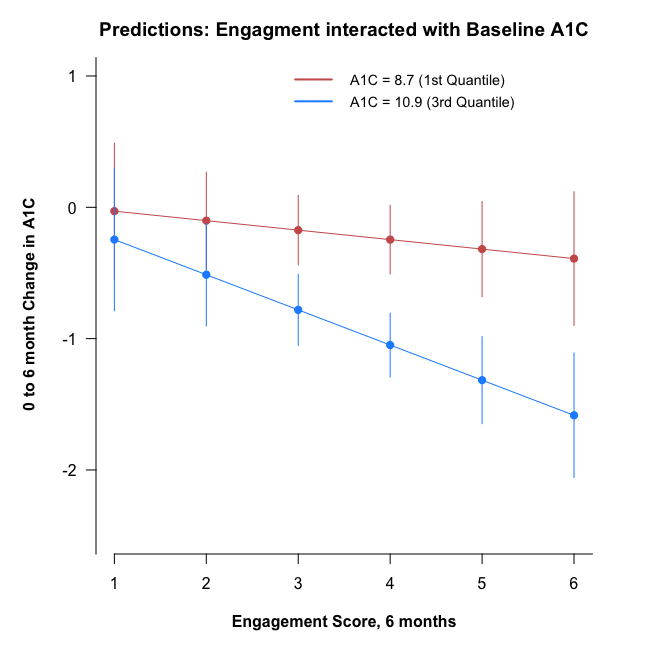


Predicted values with 95% confidence intervals for the three moderators detected: baseline depression, baseline social support, and baseline A1c. Predicted values are derived from the same OLS models, regressing 6 month change in A1c on engagement interacted with each moderator, that are reported in figure 3.
